# Synergistic effects of Chinese herbal formula combined with Microcin J25 against *Escherichia coli* and *Salmonella* in calf diarrhea and clinical evaluation of preventive and therapeutic effects in the Ningxia region

**DOI:** 10.3389/fvets.2025.1619420

**Published:** 2025-07-08

**Authors:** Dong-Zhao Ding, Lei-Xin Zhu, Qian Shao, Xin Li, Yin-Chao Tong, Wen Zhang, Yun-Peng Fan, Qi Yang, Fu-Jiang Wang, Su-Zhu Qing, Wei-Min Zhang

**Affiliations:** ^1^College of Veterinary Medicine, Northwest A&F University, Yangling, China; ^2^Veterinary Drugs and Animal Feedstuffs of Ningxia Supervision Institute, Yinchuan, China; ^3^Center for Animal Disease Prevention Control and Health Supervision, Zhongwei, China

**Keywords:** calf diarrhea, pathogen investigation, Chinese herbal formula, Microcin J25, gut microbiota

## Abstract

**Introduction:**

Calf diarrhea is one of the most common diseases causing significant economic losses in the livestock industry. This article aims to investigate the bacterial causes of calf diarrhea in the Ningxia region and to evaluate the synergistic antibacterial effect of Chinese Herbal Formula (CHF) and Microcin J25 (MccJ25) on pathogenic bacteria, as well as its clinical prevention and treatment effects on calf diarrhea.

**Methods:**

A total of 100 diarrheic fecal samples were collected from calves across 10 cattle farms in the Ningxia region. Bacterial isolation and identification were performed to detect *E. coli* and *Salmonella* strains. The *in vitro* synergistic antibacterial effects of a Chinese Herbal Formula (CHF) combined with Microcin J25 (MccJ25) were evaluated using antimicrobial susceptibility testing. Furthermore, in vivo prevention and treatment trials were conducted to assess the efficacy of the CHF-MccJ25 combination against calf diarrhea, including evaluation of clinical symptoms, fecal microbial composition, and immune parameters.

**Results:**

The results showed that out of 100 diarrheic calves fecal samples were collected from 10 cattle farms in the Ningxia Hui Autonomous Region. 97 *E. coli* strains and 20 *Salmonella* strains were detected. *In vitro*, the combination of Chinese Herbal Formula (CHF) and Microcin J25(MCJ) showed a synergistic inhibitory effect against *E. coli* and *Salmonella*. Moreover, the combined treatment also exhibited virulence-inhibitory activity. *In vivo*, the combination effectively reduced the incidence of diarrhea in healthy calves in a dose-dependence.

**Discussion:**

In summary, our study indicated that the combination of Chinese Herbal Formula (CHF) and Microcin J25(MCJ) effectively combated multidrug-resistant *E. coli* and *Salmonella*, improving both prevention and treatment of calf diarrhea by enhancing immunity and restoring gut microbiota balance.

## Introduction

Calf diarrhea is a severe disease affecting the livestock industry worldwide, particularly among neonatal calves, where it remains one of the leading causes of mortality (1). The condition is primarily triggered by pathogenic microbial infections, such as *E. coli*, *Salmonella*, and other gastrointestinal pathogens (2). Diarrhea predominantly affects calves within the first 30 days of life, and in severe cases, not only impairs normal growth and development but also adversely affects the reproductive and lactational performance of dairy cows, thereby imposing substantial economic losses on the cattle industry (3, 4).

In veterinary clinical practice, antibiotics are commonly used to treat calf diarrhea (5–7). However, the overuse and misuse of antibiotics have led to escalating antimicrobial resistance, diminishing therapeutic efficacy, and raising concerns about drug residues in animal products (8–10). More critically, long-term antibiotic use may result in recurrent diarrhea and exacerbate intestinal dysbiosis (11). Therefore, the development of safe and effective alternatives to antibiotics for managing calf diarrhea has become a focal point of current research.

Chinese Herbal Formula (CHF) has a long history in the prevention and treatment of diseases in animals. As a natural therapeutic agent, it offers advantages such as low toxicity, minimal residue, and a lack of drug resistance (12, 13). Moreover, CHF is known as antibiotic alternative, which is used to modulate physiological functions, enhance immunity, and regulate gut microbiota (14, 15), incomplete sentence, should be “making them widely used in livestock production.

Antimicrobial peptides (AMPs), representing a novel category of antibiotic substitutes, have gained considerable attention in the control of diseases in livestock and poultry (16). Microcin J25, a naturally occurring AMP, exhibits potent bactericidal activity against a variety of pathogens, particularly *E. coli* and *Salmonella* (17–19). Additionally, it has been shown to improve gut health and prevent intestinal inflammation in animals. Importantly, Microcin J25 is efficient, safe, and less prone to inducing bacterial resistance (20–23).

This study aims to explore the combined use of CHF and antimicrobial peptides as a novel strategy for the prevention and treatment of calf diarrhea.

## Materials and methods

### Samples collection

From July to August 2023, fecal samples were collected from diarrheic calves at 10 large-scale dairy or beef cattle farms located in Shizuishan, Wuzhong, Zhongwei, and Guyuan cities in Ningxia Hui Autonomous Region. A total of 100 fecal samples were collected, with 10 samples obtained from each farm (Table 1). Each sample was labeled using a unique patient ID that incorporated the initials of the sampling city in Pinyin.

**Table 1:** Basic information of the sampled cattle farms.

Sampling area	Cattle farm	Type	Breeding stock (head)	Number of samples/n
Shizuishan	1	Dairy cattle	9,000	10
Wuzhong	3	Dairy cattle	9,000	10
		Dairy cattle	1800	10
		Beef cattle	480	10
Zhongwei	1	Beef cattle	5,700	10
Guyuan	5	Beef cattle	850	10
		Beef cattle	700	10
		Beef cattle	800	10
		Beef cattle	500	10
		Beef cattle	750	10

### Isolation and identification of bacteria and detection of virulence genes

Fecal samples were 10-fold diluted in peptone buffer, and 0.2 mL of the diluted suspension was inoculated into LB broth and incubated at 37°C until the logarithmic growth phase. The cultures were then transferred onto *E. coli* chromogenic medium and *Salmonella* chromogenic medium and incubated at 37°C for 16–18 h. A single purified colony was selected using an inoculation loop for Gram staining and microscopic observation. Bacterial genomic DNA was extracted (24), and PCR amplification was performed using specific primers. The amplification system contained 3.5 μL of Taq PCR Master Mix (Dining, China), 0.5 μL each of forward and reverse primers, 0.5 μL of DNA template, and 5 μL of dd H₂O. PCR products were analyzed by 1.5% agarose gel electrophoresis (Dining, China) and visualized using a gel imaging system. Additionally, PCR was used to detect virulence genes of *E. coli* and *Salmonella*. Primers were designed based on sequences from the NCBI database. The bacterial primer sequences and annealing temperatures are shown in Table 2, and virulence gene primers are listed in Table 3. All media were purchased from Qingdao Hope Biotechnology Co., Qingdao, China. The *E. coli* strain ATCC^®^ 25922 and *Salmonella* strain ATCC^®^ 12002 preserved in our laboratory was used as a control.

**Table 2:** Sequence of 16S rDNA specific gene primers of bacteria.

Bacteria	Sequence of primer (5′-3′)	Size of product/bp	Tm/°C
*E. coli*	F: TGCCTGATGGAGGGGGATAA	776	60
	R: TTTAACCTTGCGGCCGTACT		
*Salmonella*	F: TGTAGCGGTGAAATGCGT	356	57
	R: CAGTTCCCGAAGGCACATT		

**Table 3:** Sequence of primers targeting virulence genes.

Gene	Sequence of primer (5′-3′)	Size of product/bp	Tm/°C
*β-actin*	F: CGGAAATCGTCCGTGACATC	207	58.5
	R: GAATGCCGCAGGATTCCAT		
*eaeA*	F: GTGGCGAATACTGGCGAGACT	891	61
	R: CCCCATTCTTTTTCACCGTCG		
*F41*	F: GACGGAAGGTCAACCAGG	732	57
	R: GGAGCCACCCATTCAAGT		
*STX1*	F: TGTAACTGGAAAGGTGGAGTATAC	210	57
	R: GCTATTCTGAGTCAACGAAAAATAAC		
*STX2*	F: GTTTTTCTTCGGTATCCTATTCCG	487	58
	R: GATGCATCTCTGGTCATTGTATTAC		
*STa*	F: CAACTGAATCACTTGACTCTT	158	55
	R: CTGTTCAGTCTCACGCATCAC		
*InvA*	F: TGACGGTGCGATGAAGTTT	248	58
	R: TCCGCCCCATATTATCCGT		
*sopE*	F: TGCTTCAAACGCTCCATGA	237	56
	R: CGCTTGGGCTAAAAACGTC		
*spvC*	F: AAGGTCGTTCAACAAGCC	252	59
	R: CATTTCACCACCATCACG		
*stn*	F: AGCGTTCAGGTACAGATTCAACA	341	60
	R: AAATTCGTAACCCGCTCTCGT		
*fimA*	F: AGACCGCCAGCAAATTAGTGT	321	56
	R: TGACCTCTACTATTGCGAGTCTG		

### Antibiotic susceptibility testing

Antibiotic susceptibility was determined according to the standards of the Clinical and Laboratory Standards Institute (CLSI, 2023) using the broth microdilution method. A total of nine classes of commonly used veterinary antibiotics were tested (Shanghai Xingbai Biotechnology Co., Ltd., China). The test bacterial strains and reference strains were prepared as bacterial suspensions with a final concentration of 0.5 × 10^5^ CFU/mL. Using an 8-channel pipette, 100 μL of each working solution was added to the wells of the susceptibility testing plate. Negative and positive controls were included. After incubation at 37°C for 16–18 h, results were recorded by observing the wells. The lowest concentration of the antibiotic that showed no visible bacterial growth (clear well) was recorded as the minimum inhibitory concentration (MIC).

### Checkerboard assay

The checkerboard method was used to evaluate the combined antibacterial activity of the Chinese herbal formula and Microcin J25 against *E. coli* and *Salmonella* isolated from diarrheic calves (25). Briefly, four herbal extracts—*Populus tomentosa* male flower, *Portulaca oleracea*, *Sanguisorba officinalis*, and *Euphorbia humifusa* (Sichuan Hengrui Tongda Biotechnology Co., Ltd., China)—were mixed in equal proportions to prepare the Chinese herbal formula. All four herbal extracts were prepared according to the water extraction and alcohol precipitation method specified in the Chinese Pharmacopoeia (2025 edition), with a standardized concentration of 1 g extract equivalent to 10 g crude drug. The formula and Microcin J25 (Zhongnong Yingtai Biotechnology Co., Ltd., China) were then diluted to seven gradient concentrations ranging from 1/16 MIC to 2 MIC. In a 96-well microtiter plate, each column contained a fixed concentration of drug A, and each row contained a fixed concentration of drug B. The first row and column served as monotherapy controls, and the well at the intersection (origin) served as the positive control. The initial bacterial concentration in each well was adjusted to approximately 0.5 × 10^5^ CFU/mL. *E. coli* ATCC^®^ 25,922 and *Salmonella* ATCC^®^ 12,002 were used as standard reference strains. After incubation at 37°C for 16–18 h, the results were recorded. The experiment was repeated in triplicate. FICI was calculating by given formula: FICI was calculating by given formula: FICI was calculated as the sum of the MIC of Chinese Herbal Formula in combination divided by the MIC of Chinese Herbal Formula alone, and the MIC of Microcin J25 in combination divided by the MIC of Microcin J25 alone. FICI≤0.5 means “synergy”; 0.5 < FICI≤0.75 means “partial synergy”; 0.76 < FICI≤1 denotes “additive”; 1 < FICI≤4 denotes “indifferent”; while “antagonistic” in cases which the FIC index > 4 (26).

### Growth curve assay

As previously described, the growth curve assay was performed with slight modifications (27). The growth-inhibitory effects of the Chinese herbal formula and Microcin J25, alone or in combination, against *E. coli* and *Salmonella* were evaluated by growth curve analysis. The experiment was divided into four groups: control (no treatment), Microcin J25 alone, Chinese herbal formula alone, and a combination treatment in which both Microcin J25 and the Chinese herbal formula were administered together. The concentrations used were 0.25 × MIC for single treatments and 0.25 × MIC each for the combination group. Each group was inoculated with standardized bacterial suspension (final concentration: ~0.5 × 10^5^ CFU/mL) and incubated at 37°C. Aliquots of 100 μL from each group were sampled every 2 h for 18 h, and bacterial growth was monitored by measuring the optical density at OD₆₀₀ using a microplate reader. Each experiment was performed in triplicate, and the growth curves were plotted to compare the inhibitory effects of different treatments over time.

### Determination of virulence gene downregulation

The relative expression levels of virulence genes in isolated strains were determined by real-time quantitative fluorescence PCR (RT-qPCR). RNA extraction was performed using the triazole method, with RNA concentration determined by spectrophotometry and integrity assessed via agarose gel electrophoresis. Subsequently, cDNA synthesis was carried out using an Integrated First-strand cDNA Synthesis kit, followed by RT-qPCR analysis using 2 × Fast HS SYBR QPCR Mixture. The amplification protocol was as follows: 95.0°C for 30 s; 95.0°C for 5 s, 60.0°C for 34 s, for a total of 40 cycles; followed by 95.0°C for 15 s, 60.0°C for 1 min, and 95.0°C for 15 s. The resulting qPCR data were analyzed for relative changes in gene expression levels based on the 2^−∆∆Ct^ method. Primers listed in Table 3 were utilized for this study.

### Clinical prevention trial of calf diarrhea

Eighty healthy Holstein calves aged between 7 and 14 days were randomly divided into eight groups, with ten calves per group. The groups included: a negative control group (fed with standard pasteurized milk, referred to as NC); low-, medium-, and high-dose Chinese herbal formula groups (administered at doses of 0.05 g, 0.1 g, and 0.2 g per kilogram of body weight, referred to as CHFL, CHFM, and CHFH, respectively); a Microcin J25 group (supplemented at a concentration of 0.8 g per liter of milk, referred to as MCJ); and three combination groups receiving the same doses of the Chinese herbal formula along with 0.8 g per liter of Microcin J25 (referred to as COML, COMM, and COMH, respectively). The trial lasted for 7 days. Except for the control group, all groups were supplemented with the respective agents via milk feeding twice daily. Fecal scores were defined as follows: 0 indicated firm, well-formed feces; 1, slightly loose feces; 2, unformed loose feces; and 3, watery diarrhea (28). At the end of the trial, blood and rectal swab samples were collected for further analysis.

### Clinical trial for the treatment of calf diarrhea

Twenty diarrheic Holstein calves aged between seven and 14 days were randomly divided into four treatment groups, with five calves in each group. The groups included: an enrofloxacin group (ENR), a Microcin J25 group (MCJ), a Chinese herbal formula group (CHF), and a combination group (COM). The trial lasted for 7 days. The treatment regimens were as follows: calves in the enrofloxacin group received intramuscular injections of enrofloxacin at a dose of 0.1 milliliters per kilogram of body weight once daily; the Microcin J25 group received 0.8 g of Microcin J25 per liter of milk daily; the CHF group was administered the herbal formula at 0.1 g per kilogram of body weight daily; and the combination group received both the herbal formula (0.1 g per kilogram of body weight) and Microcin J25 (0.8 g per liter of milk) daily. Fecal scores were recorded daily. Clinical resolution was defined as maintaining a fecal score below 2 for ≥2 consecutive days. At the end of the trial, blood and rectal swab samples were collected for analysis.

### 16S rDNA gene sequencing for analysis of gut microbiota

After thawing the collected calf anal swabs, microbial genomic DNA was extracted using a DNA extraction kit (Meiji Biotechnology Co., Ltd., Guangzhou, China) and detected by agarose gel electrophoresis. The V3-V4 hypervariable regions of bacterial 16S rDNA were amplified using specific primers: 341F (5′-CCTAYGGGRBGCASCAG-3′) and 806R (5′-GGACTACNNGGG TATCTAAT-3′). PCR products were purified with magnetic beads, pooled based on concentration, and target bands were excised for library construction. The constructed libraries were quantified using Qubit® and RT-qPCR. Qualified libraries were sequenced on the Illumina MiSeq platform (PE250 mode, Novaseq 6,000 system). Sample data were demultiplexed based on barcode and primer sequences. After removing barcodes and primers, paired-end reads were merged using FLASH to generate raw Tags. Reverse primer sequences were trimmed with Cutadapt to minimize interference in downstream analyses. Raw Tags were rigorously filtered with fastp to obtain high-quality Tags, which were then aligned against a reference database for chimera removal, yielding final effective OTUs. The validated OTUs were subjected to clustering analysis. Subsequent analyses included OTUs abundance profiling, alpha diversity (within-sample diversity), beta diversity (between-sample diversity), and taxonomic composition.

### Statistical analysis

All experimental data were analyzed and visualized using GraphPad Prism 8. In all comparisons, *p* < 0.01 or *p* < 0.05 was considered statistically significant.

## Results

### Isolates of *E. coli* and *Salmonella* from the samples

**Figure 1:**
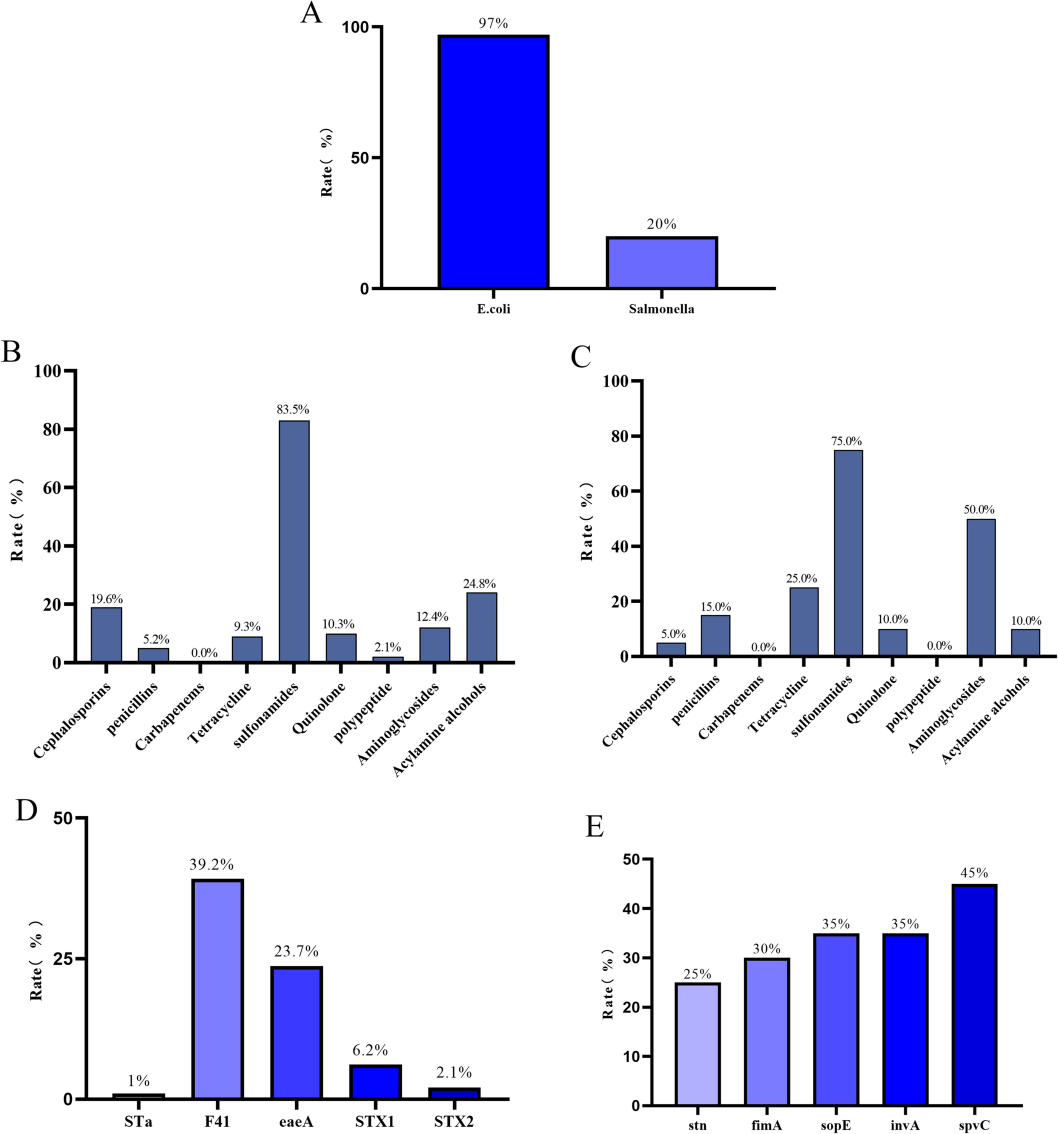
**(A)** Isolation rate of *E. coli* and *Salmonella*.**(B)** Antibiotic resistance rates of 97 identified *E. coli* strains against 9 antibiotic classes.**(C)** Antibiotic resistance rates of 20 identified *Salmonella* strains against 9 antibiotic classes.**(D)** Detection rates of 5 virulence genes in 97 identified *E. coli* strains.**(E)** Detection rates of 5 virulence genes in 20 identified *Salmonella* strains.

Among the 100 fecal samples collected from diarrheic calves, a total of 97 *E. coli* and 20 *Salmonella* isolates were recovered (Figure 1A). All 97 *E. coli* isolates were sensitive to carbapenems. The highest resistance rate was observed against sulfonamides (83.5%), followed by resistance to amphenicols (24.8%), cephalosporins (19.6%), aminoglycosides (12.4%), quinolones (10.3%), tetracyclines (9.3%), penicillins (5.2%), and polymyxins (2.1%) (Figure 1B). All 20 *Salmonella* isolates were also sensitive to carbapenems and polymyxins, but exhibited the highest resistance to sulfonamides (75.0%), followed by aminoglycosides (50.0%), tetracyclines (25.0%), penicillins (15.0%), quinolones (10.0%), amphenicols (10.0%), and cephalosporins (5.0%) (Figure 1C).

Among the *E. coli* isolates, the *F41* virulence gene had the highest detection rate (39.2%), followed by *eaeA* (23.7%), *STX1* (6.2%), *STX2* (2.1%), and *STa* (1%) (Figure 1D). For *Salmonella*, the most frequently detected virulence gene was *spvC* (45%), followed by *sopE* and *invA* (both 35%), *fimA* (30%), and *stn* (25%) (Figure 1E).

### Synergistic antibacterial assay

**Figure 2:**
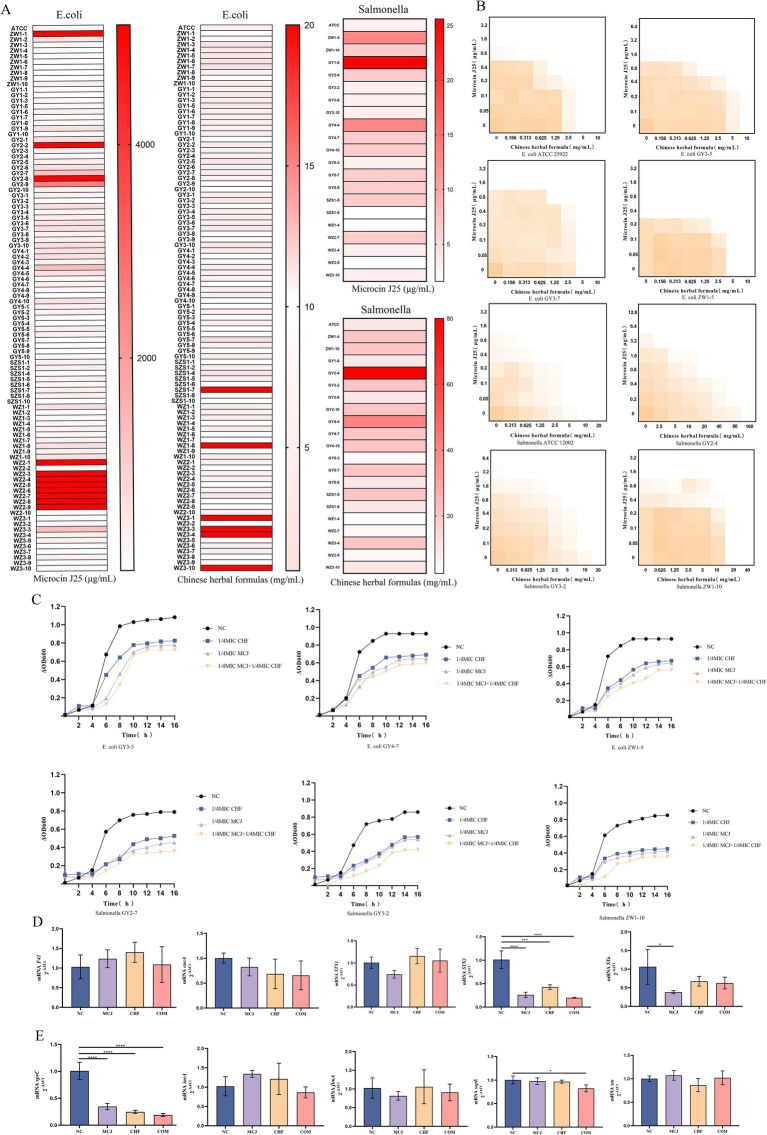
**(A)** MIC of Chinese herbal formulas and Microcin J25 against the isolated *E. coli* and *Salmonella* strains.**(B)** Synergistic antibacterial effect determined by the checkerboard method.**(C)** Inhibition of growth in *E. coli* and *Salmonella* by Chinese herbal formulas and Microcin J25.**(D)** Effect of Chinese herbal formulas and Microcin J25 on the relative mRNA expression of virulence genes in *E. coli*.**(E)** Effect of Chinese herbal formulas and Microcin J25 on the relative mRNA expression of virulence genes in *Salmonella*. All data were expressed as mean ± SD values with three independent experiments performed in triplicate.^*^p < 0.05; ^***^p < 0.001; ^****^p < 0.0001.

To evaluate the potential synergistic effect of the Chinese herbal formula in combination with Microcin J25, the minimum inhibitory concentrations (MIC) of each agent against 97 previously identified *E. coli* and 20 *Salmonella* isolates were determined. Subsequently, checkerboard assays and bacterial growth curve analyses were conducted on three *E. coli* strains (GY3-5E, GY3-7E, ZW1-5E) and three *Salmonella* strains (GY3-2S, GY2-4S, ZW1-10S) harboring multiple virulence genes. The MIC of individual agents are shown in Figure 2A. As illustrated in Figure 2B and detailed in Table 4, the combination of the Chinese herbal formula with Microcin J25 exhibited partial synergy against the *E. coli* reference strain and either partial synergy or additive effects against the remaining *E. coli* isolates. For *Salmonella*, synergistic effects were observed against the reference strain, while partial synergy or indifferent interactions were observed for the clinical isolates.

**Table 4:** FICI values of Chinese herbal formulas combined with Microcin J25.

Strain	FICI	Outcome
ATCC® 25,922	0.75	Partial Synergy
GY3-5E	0.75	Partial Synergy
GY3-7E	0.75	Partial Synergy
ZW1-5E	1.5	Indifference
ATCC® 12,002	0.25	Synergy
ZW1-10S	1.25	Indifference
GY2-4S	1.25	Indifference
GY3-2S	0.75	Partial Synergy

Importantly, as shown in Figure 2C, the combination treatment consistently suppressed the growth of both *E. coli* and *Salmonella*. In *E. coli*, no significant changes were observed in the mRNA expression levels of *F41*, *eaeA*, and *STX1* (*p* > 0.05). However, the expression of *STX2* was significantly reduced in the Microcin J25, Chinese herbal formula, and combination groups compared with the control group (*p* < 0.001), with the most pronounced inhibition observed in the combination group. In addition, the expression of *STa* was significantly reduced in the Microcin J25 group compared with the control (*p* < 0.05) (Figure 2D). For *Salmonella*, the expression levels of *stn*, *fimA*, and *invA* were unchanged following treatment (*p* > 0.05). The expression of *sopE* was significantly downregulated in the combination group compared to the control (*p* < 0.05), and *spvC* expression was markedly decreased in the Microcin J25, Chinese herbal formula, and combination groups (*p* < 0.0001), with the greatest effect observed in the combination treatment (Figure 2E).

### Effects of combined application on fecal scores, diarrhea, and immunoglobulins of healthy calves

**Figure 3:**
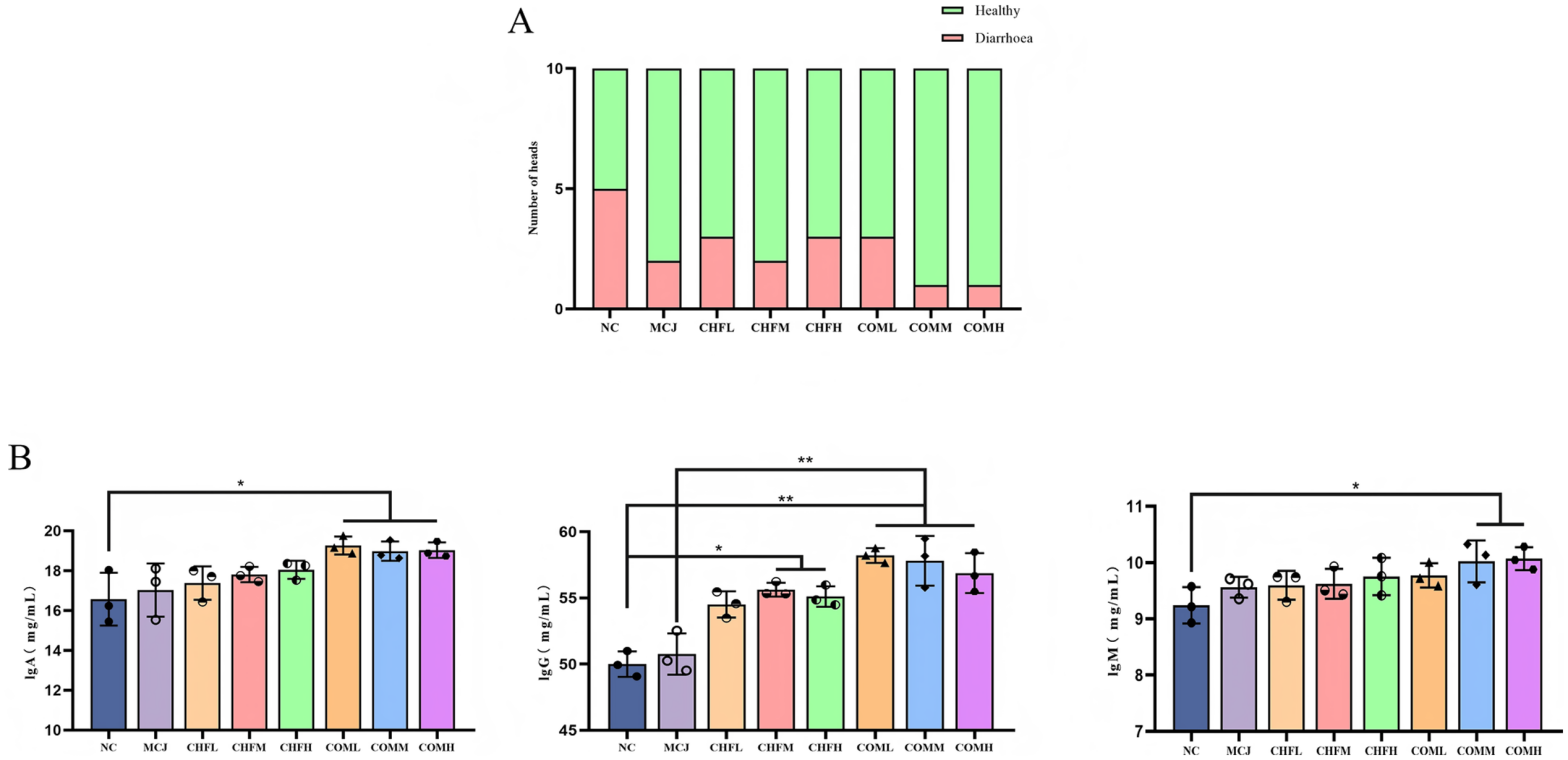
Effects of Chinese herbal formulas combined with Microcin J25 on diarrhea incidence and immunoglobulin levels in healthy calves. **(A)** Incidence of diarrhea in healthy calves treated with different groups. **(B)** Serum levels of IgA, IgG, and IgM in calves treated with different combinations. All data are expressed as mean ± SD. ^*^p < 0.05; ^**^p < 0.01. NC, Negative control group; MCJ, Calves treated with Microcin J25; CHFL, CHFM, CHFH, Calves treated with low, medium, and high-dose Chinese herbal formula. COML, COMM, COMH, Calves treated with low, medium, and high-dose Chinese herbal formula combined with Microcin J25, respectively.

As shown in Table 5, all treatment groups exhibited lower fecal scores compared to the NC group. On day 5, the fecal scores in the MCJ, CHFM, CHFH, COML, COMM, and COMH groups were significantly lower than those in the NC group (*p* < 0.05). The highest diarrhea incidence was observed in the NC group at 50%, whereas the MCJ group showed a marked reduction to 20%. The diarrhea rates in the CHFL, CHFM, and CHFH groups were also reduced compared to the NC group, with the CHFM group showing the best effect at 20%. A further reduction in diarrhea incidence was observed in the combination groups, particularly in the COMM and COMH groups, which showed the lowest incidence at 10% (Figure 3A).

**Table 5:** Effects of Chinese herbal formulas and Microcin J25 on fecal score of calves.

Days(d)	Fecal scores of each group							
	NC	MCJ	CHFL	CHFM	CHFH	COML	COMM	COMH
0	1.1 ± 0.49	1.2 ± 0.42	1.1 ± 0.52	1.1 ± 0.49	1.2 ± 0.48	1.2 ± 0.49	1.1 ± 0.69	1.0 ± 0.52
1	1.2 ± 0.42	1.1 ± 0.32	1.1 ± 0.32	1.1 ± 0.32	1.2 ± 0.42	1.2 ± 0.49	1.2 ± 0.49	1.1 ± 0.75
2	1.3 ± 0.48	1.1 ± 0.32	1.1 ± 0.32	1.3 ± 0.48	1.3 ± 0.67	1.5 ± 0.69	1.2 ± 0.49	1.6 ± 0.82
3	1.4 ± 0.48	1.3 ± 0.67	1.2 ± 0.42	1.3 ± 0.48	1.2 ± 0.42	1.5 ± 0.53	1.5 ± 0.53	1.6 ± 0.52
4	1.8 ± 0.92	1.2 ± 0.32	1.6 ± 0.70	1.4 ± 0.70	1.6 ± 0.84	1.3 ± 0.49	1.2 ± 0.48	1.3 ± 0.82
5	2.0 ± 0.84	1.3 ± 0.48^*^	1.5 ± 0.53	1.3 ± 0.51^*^	1.4 ± 0.41^*^	1.3 ± 0.49^*^	1.2 ± 0.53^*^	1.2 ± 0.63^*^
6	1.7 ± 0.82	1.5 ± 0.71	1.5 ± 0.53	1.5 ± 0.53	1.5 ± 0.53	1.5 ± 0.78	1.4 ± 0.35	1.6 ± 0.51
7	1.7 ± 0.82	1.4 ± 0.52	1.7 ± 0.67	1.3 ± 0.48	1.7 ± 0.48	1.1 ± 0.38	1.4 ± 0.76	1.3 ± 0.51

All data are expressed as mean ± SD. *P<0.05. NC, Negative control group; MCJ, Calves treated with Microcin J25; CHFL, CHFM, CHFH, Calves treated with low, medium, and high-dose Chinese herbal formula. COML, COMM, COMH, Calves treated with low, medium, and high-dose Chinese herbal formula combined with Microcin J25, respectively.

Serum IgA levels in the COML, COMM, and COMH groups were significantly higher than those in the NC group (*p* < 0.05), while no significant changes were found in the MCJ, CHFL, CHFM, or CHFH groups (*p* > 0.05). Serum IgM levels were significantly elevated in the COMM and COMH groups compared to the NC group (*p* < 0.05), whereas no significant differences were observed in the MCJ, CHFL, CHFM, CHFH, or COML groups (*p* > 0.05). Serum IgG levels were significantly increased in the CHFM and CHFH groups (*p* < 0.05), and were markedly elevated in the COML, COMM, and COMH groups compared to both the NC and MCJ groups (*p* < 0.01) (Figure 3B).

### Effects of combined application on the gut microbiota of healthy calves

**Figure 4:**
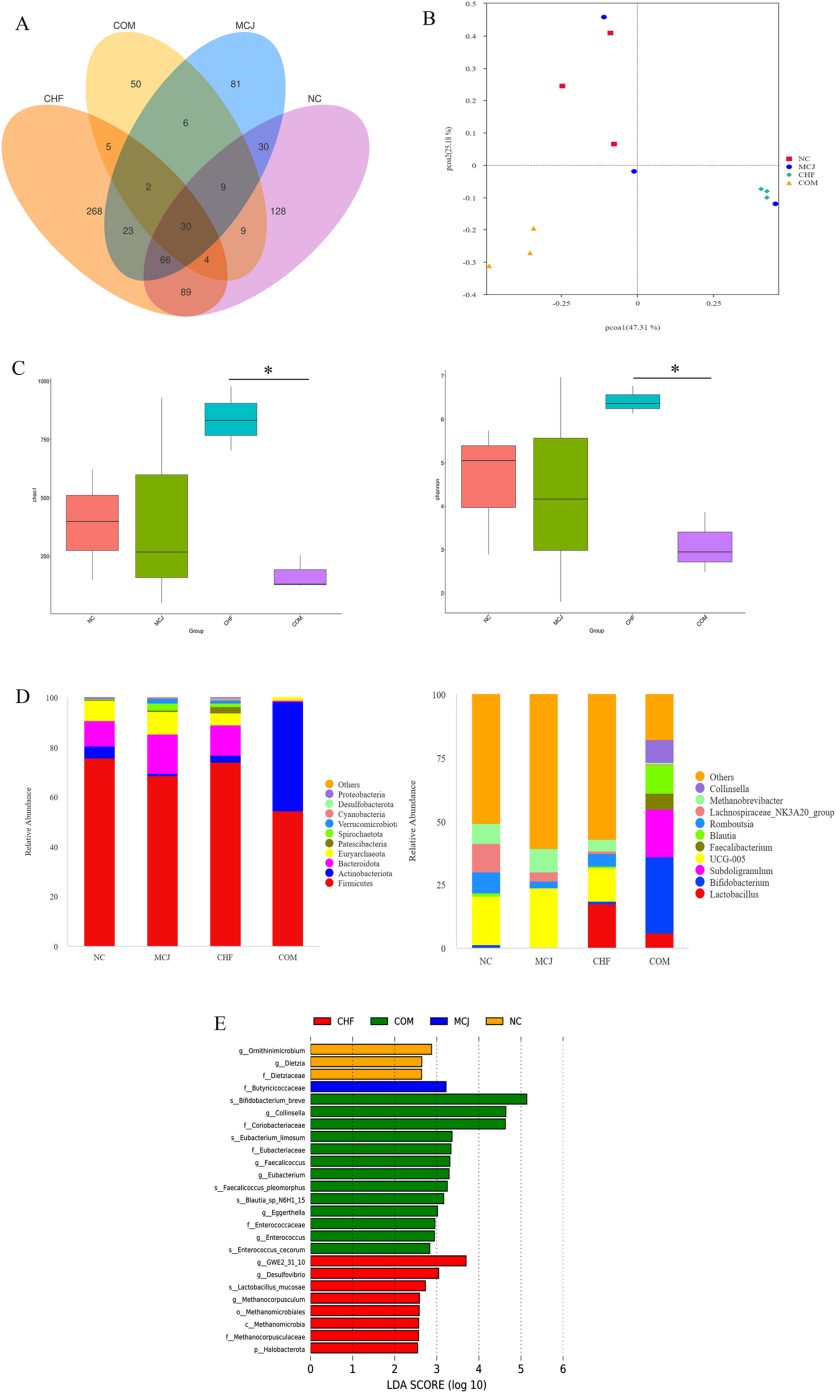
Comparison of gut microbiota structure and distribution across different treatment groups. **(A)** Venn diagram showing the overlap of OTUs between different groups. **(B)** Beta diversity analysis. **(C)** Alpha diversity analysis. **(D)** Relative abundance of microbiota at the phylum and genus levels. **(E)** Linear discriminant analysis (LDA) was used to identify the most differentially abundant bacterial taxa among groups, with only those having an LDA score >3.0 shown. ^*^p < 0.05. NC, Negative control group; MCJ, Calves treated with Microcin J25; CHF, Calves treated with Chinese herbal formula. COM, Calves treated with Chinese herbal formula combined with Microcin J25.

Based on the above experimental results, the CHFM and COMM groups, which showed the best performance, were selected along with the NC and MCJ groups for 16S rDNA sequencing to explore their regulatory effects on the gut microbiota. As shown in Figure 4A, the Venn diagram of OTUs revealed that the CHF group had 268 OTUs, followed by the NC group with 128, the MCJ group with 81, and the COM group with 50. Figure 4B indicates that the sample points of the NC group were farther from those of the CHF and COM groups in the PCoA analysis, suggesting significant differences in microbial structure. The Chao1 and Shannon indices in the COM group were significantly decreased, while those in the CHF group increased significantly (*p* < 0.05), as shown in Figure 4C. At the phylum level (Figure 4D), *Firmicutes* was the dominant phylum in the NC, MCJ, and CHF groups, while both *Firmicutes* and *Actinobacteriota* dominated in the COM group. *UCG-005* was the dominant genus in the NC and MCJ groups, *Lactobacillus* in the CHF group, and *Bifidobacterium* in the COM group. LEfSe analysis (Figure 4E) further showed that *Lactobacillus mucosae*, *Methanocorpusculum*, and *Methanocorpusculaceae* were significantly enriched in the CHF group. Meanwhile, *Bifidobacterium breve* and *Collinsella* were enriched in the COM group. These findings indicate that CHF improved gut microbiota diversity and stability in calves, increasing the abundance of beneficial bacteria, while COM mainly enhanced the abundance of specific probiotics.

### Effect of combination application on cure rate, recovery time, and cytokine levels of diarrheic calves

**Figure 5:**
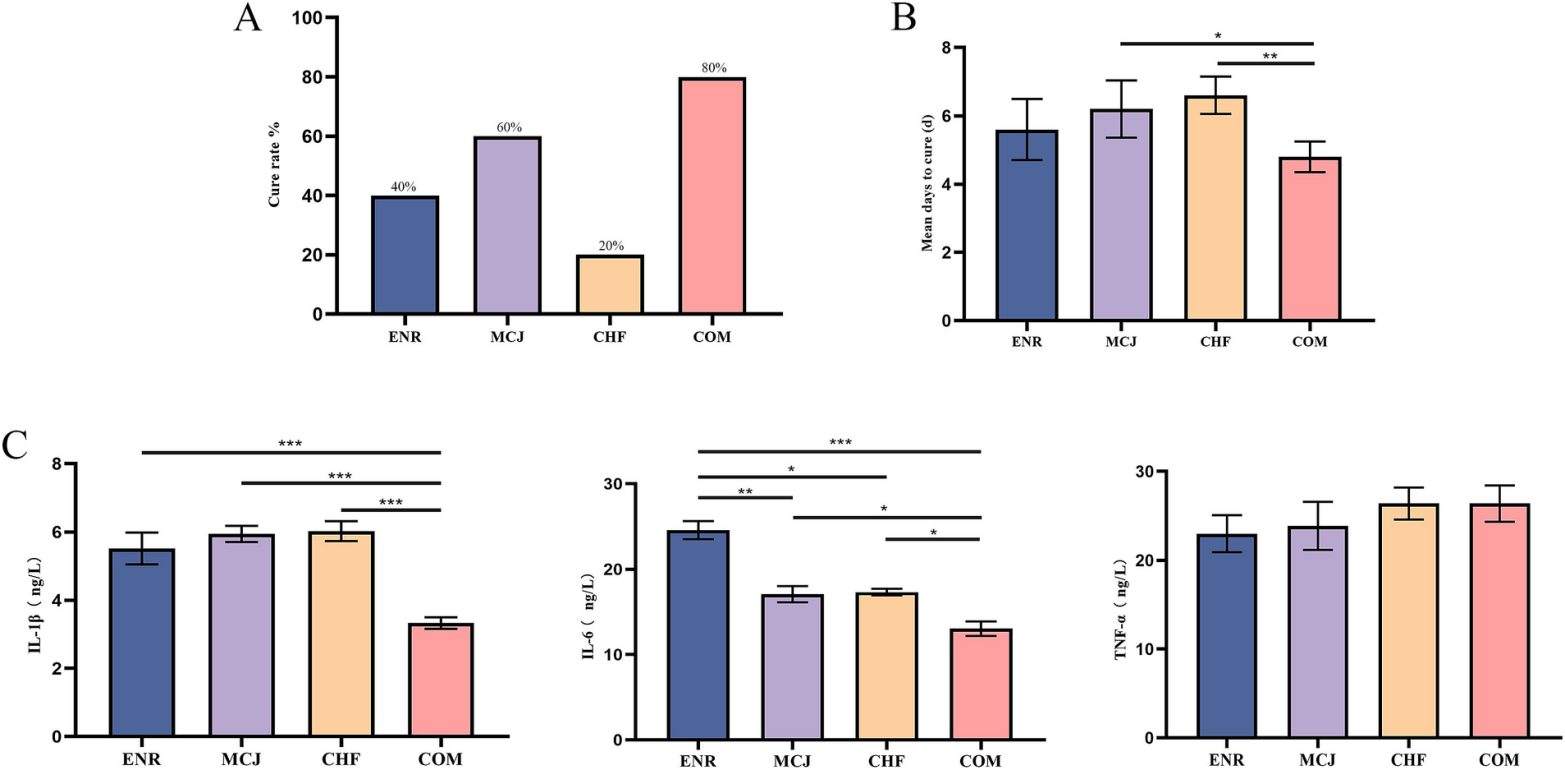
Effects of Chinese herbal formulas combined with Microcin J25 on cure rate, recovery time, and inflammatory cytokines in diarrheic calves. **(A)** Cure rates of calves in different treatment groups. **(B)** Mean days to recovery in each group, showing the number of days required for recovery. **(C)** Serum levels of proinflammatory cytokines: IL-1*β*, IL-6, and TNF-*α* in different treatment groups. ^*^p < 0.05; ^**^p < 0.01; ^***^p < 0.001. ENR, Calves treated with enrofloxacin; MCJ, Calves treated with Microcin J25 alone; CHF, Calves treated with Chinese herbal formulas alone; COM, Calves treated with a combination of Chinese herbal formulas and Microcin J25.

The cure rate was lowest in the CHF group at 20%, followed by the ENR group at 40%, while the COM group exhibited the highest cure rate at 80% (Figure 5A). The COM group showed a significantly shorter average recovery time than the MCJ group (*p* < 0.05) and a markedly shorter time than the CHF group (*p* < 0.01), indicating superior therapeutic efficacy (Figure 5B). Moreover, the COM group had significantly lower serum IL-1β and IL-6 levels compared to the other groups (*p* < 0.05) (Figure 5C).

### Effect of combination application on the gut microbiota of diarrheic calves

**Figure 6:**
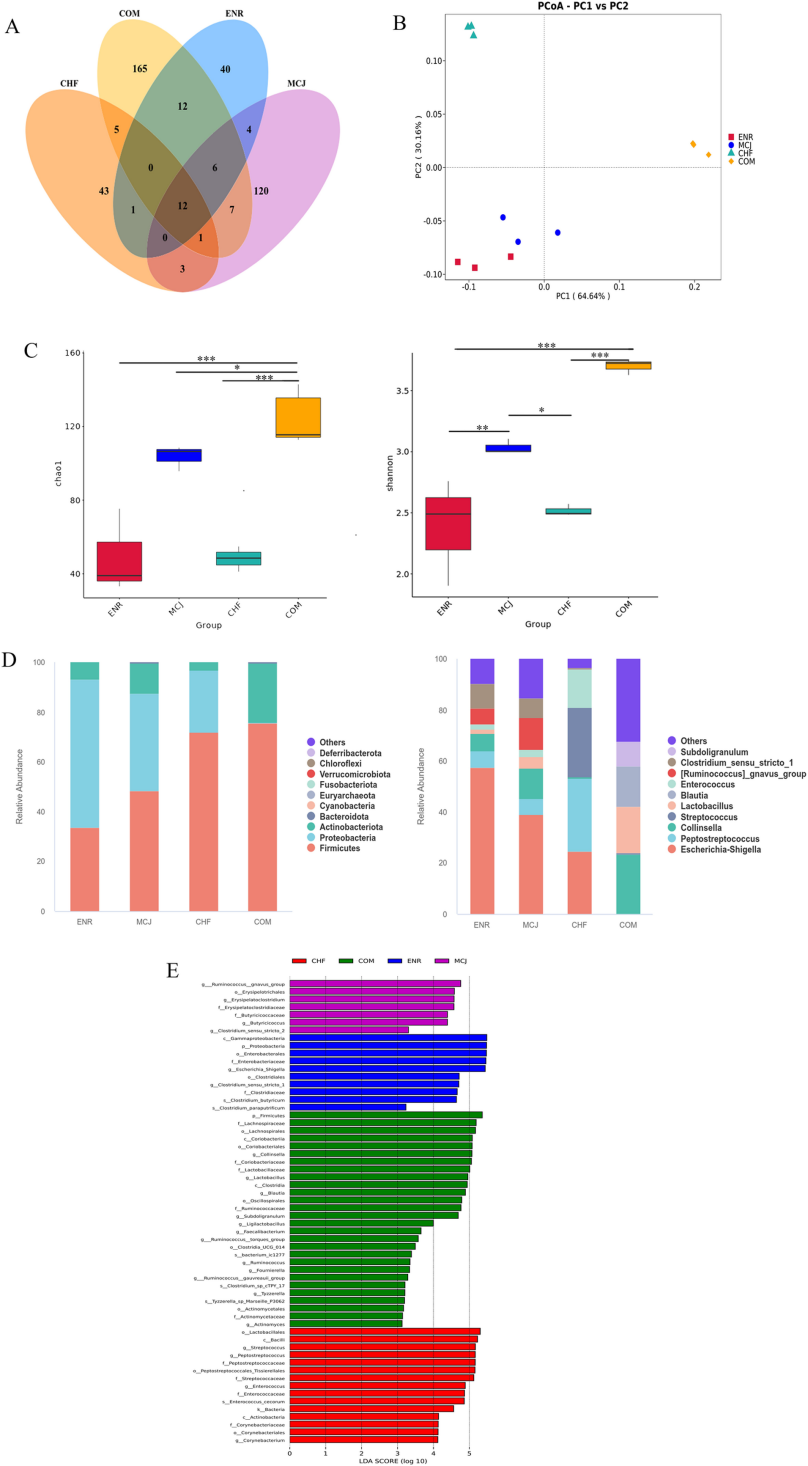
Comparison of gut microbiota structure and distribution across different treatment groups. **(A)** Venn diagram showing the overlap of OTUs between different groups. **(B)** Beta diversity analysis. **(C)** Alpha diversity analysis. **(D)** Relative abundance of microbiota at the phylum and genus levels. **(E)** Linear discriminant analysis (LDA) was used to identify the most differentially abundant bacterial taxa among groups, with only those having an LDA score >3.0 shown. ^*^p < 0.05; ^**^p < 0.01; ^***^p < 0.001. ENR, Calves treated with enrofloxacin; MCJ, Calves treated with Microcin J25 alone; CHF, Calves treated with Chinese herbal formulas alone; COM, Calves treated with a combination of Chinese herbal formulas and Microcin J25.

As shown in the Venn diagram (Figure 6A), the number of OTUs detected in the ENR, CHF, MCJ, and COM groups was 40, 43, 120, and 165, respectively. The COM group exhibited a distinct scatter distribution from the ENR and MCJ groups in PCoA analysis, indicating substantial differences in microbial community structures between these groups (Figure 6B). Compared to the other three groups, the COM group showed significantly higher Chao1 and Shannon indices (*p* < 0.05), suggesting increased richness and diversity (Figure 6C).

At the phylum level, *Firmicutes* was the dominant phylum in both the CHF and COM groups, while *Firmicutes* and *Proteobacteria* were dominant in the ENR group. At the genus level, *Escherichia-Shigella* was predominant in the ENR and MCJ groups, while *Enterococcus*, *Streptococcus*, and *Peptostreptococcus* were dominant in the CHF group. In contrast, *Collinsella*, *Lactobacillus*, and *Blautia* were enriched in the COM group (Figure 6D). LEfSe analysis further revealed higher abundance of *Escherichia-Shigella* and *Enterococcus* in the ENR group; increased *Ruminococcus*, *Blautia*, and *Clostridium* in the MCJ group; elevated *Lactobacillus*, *Bacillus*, and *Peptostreptococcus* in the CHF group; and increased *Lactobacillus*, *Bifidobacterium*, and *Clostridium* in the COM group (Figure 6E).

These findings indicate that the combination of Chinese herbal formula and Microcin J25 effectively reduced the abundance of harmful bacteria while enriching beneficial microbes in the gut of diarrheic calves, thereby improving the overall microbial structure.

### Correlation analysis of intestinal flora, anti-inflammatory factors, and immunoglobulins

**Figure 7:**
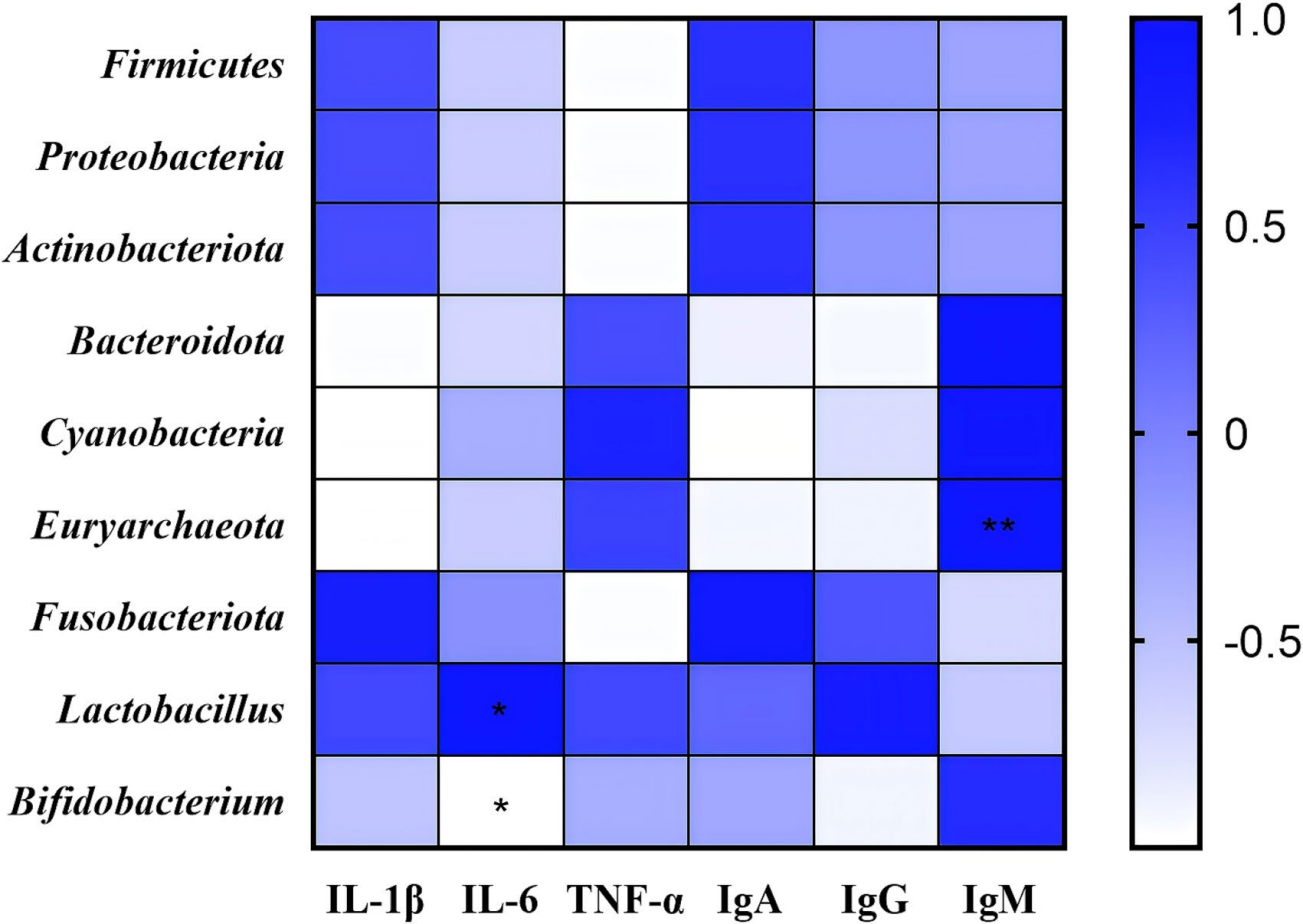
The heatmap illustrates Pearson correlation coefficients among anti-inflammatory factors, immunoglobulins, and gut microbiota. *p < 0.05, **p < 0.01. n = 3.

Subsequently, correlation analyses were performed among anti-inflammatory factors, immunoglobulins, and dominant bacterial taxa detected in rectal swabs (Figure 7). The abundance of *Euryarchaeota* showed a highly significant positive correlation with serum IgM levels (*p* < 0.01). A significant positive correlation was observed between *Lactobacillus* abundance and serum IL-6 levels (*p* < 0.05), whereas *Bifidobacterium* abundance exhibited a significant negative correlation with serum IL-6 levels (*p* < 0.05). These findings suggest that the combined treatment of Chinese herbal formula and Microcin J25 may improve anti-inflammatory status and immune function in calves through modulation of gut microbiota composition.

## Discussion

Diarrhea is one of the most common gastrointestinal diseases in calves, causing significant economic losses to the global livestock industry (29). Infectious diarrhea remains a leading cause of morbidity and mortality in neonatal calves (30). The most prevalent pathogens involved in neonatal calf diarrhea include bacteria (such as *E. coli* and *Salmonella*), viruses (such as rotavirus and coronavirus), and protozoa (such as *Cryptosporidium*) (31, 32). Bacterial infections, particularly those caused by pathogenic *E. coli* and *Salmonella*, are considered major contributors to calf diarrhea outbreaks in farms. In this study, 100 fecal samples from diarrheic calves were collected from four cities in Ningxia, from which 97 *E. coli* and 20 *Salmonella* isolates were identified. Most of these isolates exhibited high resistance to sulfonamides, suggesting that sulfonamide antibiotics may be ineffective for treating calf diarrhea in this region. Reducing antibiotic misuse and enhancing surveillance of resistant bacterial strains are essential strategies for improving calf health (33). Additionally, virulence gene analysis showed high detection rates of *E. coli* F41 and *Salmonella spvC*, indicating that strains carrying these genes may possess strong pathogenicity.

Due to the reduced efficacy of antibiotics in treating calf diarrhea, alternative strategies are urgently needed (34). Antimicrobial peptides, such as Microcin J25, show strong antimicrobial activity, immunomodulatory effects, and gut microbiota regulation potential (19, 35–40). However, their clinical application remains limited. The Chinese herbal formula used in this study, composed of *Populus tomentosa* male flower, *Portulaca oleracea*, *Euphorbia humifusa*, and *Sanguisorba officinalis*, contains bioactive flavonoids, polysaccharides, and organic acids with known anti-inflammatory and gut-protective effects (41–45). We demonstrated that herbal formula combined with Microcin J25 showed partial or full synergy against *E. coli* and *Salmonella in vitro*. The combination more effectively inhibited bacterial growth than either agent alone and significantly downregulated the expression of key virulence genes, including *STX2* in *E. coli* and *spvC* in *Salmonella*, consider citing specific mechanisms from literature. This mechanism may be related to the destruction of bacterial biofilms. Relevant research reports indicate that the combined use of Chinese herbal medicines and polypeptide antibacterial substances triggers synergistic antibacterial effects by increasing cell membrane permeability, promoting intracellular ROS production, and inhibiting the expression of the mcr-1 gene (46).

Calf diarrhea disrupts intestinal microbiota and reduces immunity, making microbiota regulation and immune enhancement key to prevention (29, 47). Previous studies have shown that both Chinese herbal formulas and Microcin J25 improve gut health and reduce diarrhea in animals (48–50), though their combined use has not been reported. Based on in vitro synergy, we hypothesized that their combination could also be effective *in vivo*. As diarrhea is common in calves under 14 days, we selected seven-to fourteen-day-old calves to avoid colostrum influence. The combined treatment (COM) group showed the lowest diarrhea rates at 10%, with COMM and COMH being most effective, indicating strong preventive potential. Further trials are needed to clarify dose-dependence. Immunoglobulins are key indicators of humoral immunity (51). IgA protects mucosal surfaces, IgG mediates specific immune responses, and IgM is the first antibody produced after infection (52). CHF significantly increased serum immunoglobulins, and COM showed even greater improvement. Given the immature gut microbiota of neonatal calves (53), maintaining microbial diversity is critical. 16S rDNA sequencing revealed that CHF alone significantly increased Chao1 and Shannon indices, improving microbial diversity. *Firmicutes* dominated in healthy calves, consistent with previous studies (54). While COM increased beneficial bacteria, diversity declined, possibly due to enhanced antimicrobial synergy. Thus, COM may suit acute-phase intervention, while CHF alone is preferable for long-term gut stability.

Currently, large-scale cattle farms primarily rely on broad-spectrum antibiotics to treat calf diarrhea. However, such treatments can disrupt the gut microbiota, leading to a reduction in beneficial bacteria, an increase in resistant strains, and drug residues (55). In this study, we found that the cure rate in the COM group reached 80%, significantly higher than enrofloxacin, Microcin J25, or the herbal formula alone. Additionally, the COM group had a shorter recovery time compared to the ENR group. Proinflammatory cytokines such as IL-1β, IL-6, and TNF-*α* are key mediators of immune and inflammatory responses and are closely associated with the pathogenesis of diarrhea. When the intestinal barrier is compromised, pathogens can invade and trigger inflammation (56–59). Calves in the COM group showed significantly lower levels of IL-1β and IL-6, suggesting that the combination therapy effectively alleviated intestinal inflammation. Gut microbiota plays a vital role in both the onset and resolution of diarrhea (60). The COM group showed significantly higher Chao1 and Shannon indices than the other groups, indicating improved microbial richness and diversity. Diarrheic calves typically exhibit an overgrowth of Proteobacteria, a marker of dysbiosis and diarrhea risk (61). In the COM group, the relative abundance of *Firmicutes*, *Lactobacillus*, and *Bifidobacterium* increased, while *Proteobacteria* and *Escherichia-Shigella* decreased. This suggests that the combination treatment promoted beneficial bacteria, suppressed harmful bacteria, and helped restore microbial balance (62). In contrast, the ENR group showed higher levels of *Proteobacteria* and *Escherichia-Shigella*, indicating potential microbiota disruption due to prolonged antibiotic use. Furthermore, the abundance of *Euryarchaeota* was extremely significantly positively correlated with the level of serum IgM. The abundance of *Bifidobacterium* was significantly negatively correlated with the level of serum IL-6. This indicates that the traditional Chinese herbal formula and Microcin J25 may enhance the immune function and anti-inflammatory ability of calves by regulating the intestinal flora.

In conclusion, our study showed a high prevalence of *E. coli*, *Salmonella*, and their associated virulence genes in diarrheic calves in the Ningxia region, which warrants attention. Notably, the combination of Chinese herbal formulas and Microcin J25 exhibited synergistic antibacterial activity against *E. coli* and *Salmonella*. Clinically, this combination reduced diarrhea incidence, improved cure rates, enhanced immunity, alleviated inflammatory responses, and helped regulate gut microbiota composition in calves. These findings highlight the great potential of Chinese herbal medicine and antimicrobial peptides in the prevention and treatment of calf diarrhea.

## Data Availability

The data presented in the study are deposited in the NCBI repository, accession number PRJNA1281273.
